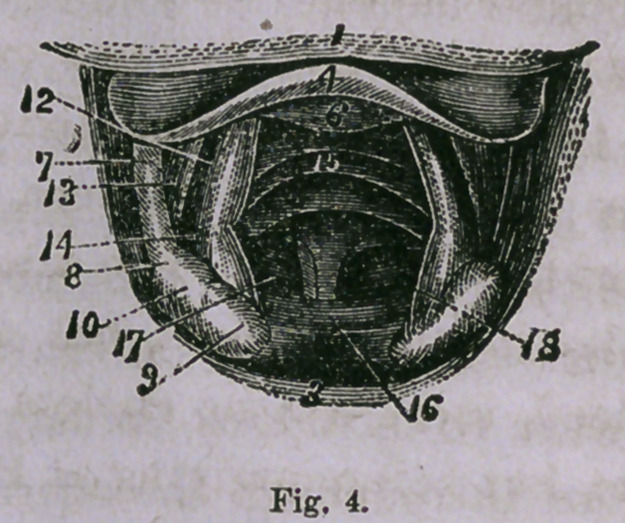# Practical Papers on Diseases of the Throat and Air Passages

**Published:** 1866-09

**Authors:** Edward B. Stevens

**Affiliations:** Professor of Materia Medica in the Miami Medical College of Cincinnati


### Miscellaneous.

### Practical Papers on Diseases of the Throat and Air Passages.

BY EDWARD B. STEVENS, M. D.,

Professor of Materia Medica in the Miami Medical College of Cincinnati.

*The Laryngoscope.*—One of the most notable features of our current medical progress is seen in the ingenious and valuable improvements in so many departments of special diagnosis. Many fields of inquiry heretofore necessarily vague and uncertain, and hence unsatisfactory and embarrassing to the practitioner, are now reduced to the definite comfort of exact science. These more positive and exact modes of inquiry are contributing in their various departments very largely to the character and usefulness of our general profession. Take for illustration the advance in ophthalmological science developed with the successful introduction of the ophthalmoscope. Auscultation and percussion have revolutionized the diagnosis of thoracic diseases. Just now we have amongst the recent additions to science the use of the endoscope. All these are rapidly successive steps in the progress of the exact diagnosis of disease, enabling us to grasp the nature of obscure affections and determine their treatment more promptly and satisfactorily than ever heretofore.

As with most all practically useful improvements and inventions, we wonder that so simple an expedient as the laryngoscope should have remained unsuggested heretofore, and we hardly refrain from a feeling of vexation that the early efforts in that direction were so obstinately rejected for a whole century.

The object to be attained is to throw light upon and obtain a view of structures absolutely without the range of any direct line of vision. As Dr. Mackenzie very well states it, "the only principle concerned in the art of laryngoscopy is the optical law, that when rays of light fall on a plane surface, the angle of reflection is equal to the angle of incidence. A small mirror is placed at the back of the throat, at such an inclination that luminous rays falling on it are projected into the cavity of the larynx. At the same time the image of the interior of the larynx (lighted up by the luminous rays) is formed on the mirror and seen by the observer."

To accomplish what seems so ready and feasible to us now, was undertaken in 1743 by a distinguished Frenchman, M. Levret, who used various ingenious instrumental devices for reaching polypoid growths, etc., in the throat and nostrils. He used some form of speculum; and it is quite probable that the dentist's mirror has been used for exploring hidden parts of the cavity of the mouth and throat from time immemorial.

Bozzini appears in 1807 to have been on the right track, and in 1829, Dr. Babbington of London, exhibited a plan of inspecting the larynx by a series of mirrors and reflected light very closely resembling the laryngoscope now in use. Successively various improvements and advances were made toward the solution of the difficulty proposed, until in 1857, Prof. Czermak, of Pesth, developed and perfected the simple mechanical contrivances now employed by many enthusiastic manipulators all over the medical world. Various claims to priority are preferred in this discovery, some of them doubtless with merit, but Czermak fairly receives the credit of perfecting the mechanism and indeed of creating the art of laryngoscopy. Such of our readers as desire to follow up the literature of this discovery may read with interest the little book of Dr. Morell Mackenzie. So much of the *history*, however, of an invention about which considerable has already been said in this journal, we thought due to our readers.

For performing laryngoscopy, ordinarily, there are only required three elements of mechanism, although different operators employ a great variety of extra contrivance adjuvant; indeed each individual soon learns to adapt himself to special and peculiar devices to meet his own peculiar views and expertness.

1st.—The *Laryngeal Mirror*, ordinarily an oval or circular mirror, of polished metal, or glass backed with amalgam and protected with metal, about eight-tenths of an inch in diameter, but not uniform in size. This mirror is fixed to a handle or shank, at a convenient angle or curve for introduction into the posterior fauces.

2d.—*The Illuminator.*—Sunlight may be used under favorable circumstances, but usually artificial light will be found more satisfactory and manageable. Ordinary gas light from an argand burner answers a good purpose; a kerosene lamp is employed by many, and various lamps and lanterns have been contrived by different laryngoscopists. These are unnecessary now to detail, as we only desire to make the principal and general plan of operating readily understood by our readers. It is frequently found of advantage to use apparatus, as lenses or otherwise, for concentrating the light.

Now with whatever means of illumination you resort to, you may arrange to throw the ray of light *direct* upon the plane of the laryngeal mirror, or as is more generally practiced, you have

3d.—*The Reflector.*—Ruete's ophthalmoscopic mirror seems to have afforded the first suggestion as to a convenient reflector for laryngoscopic purposes. Czermak used it first for concentrating luminous rays, and a modification of this mechanism is still retained. Some attach the mirror to a spectacle frame, the mirror having a central perforation, the operator looks directly through it on the laryngeal mirror. Some attach the reflector to a band passing round the head. The first is Semeleder's plan; the frontal band is the device of Kramer.

With these brief explanations the reader is prepared to understand with but little comment the following wood cut illustration of a laryngoscopic examination, which we are courteously permitted to copy from the last edition of Dr. Bennett's Practice of Medicine, published by Messrs. Wood & Co. of New York.

You observe the laryngeal mirror is held in position by the operator using for this purpose his right hand, depressing the tongue with the common tongue depressor held in his left hand. The gas jet is placed a little to one side and to the rear of the patient's face, the glare being screened from the observer by a mounted shade. In this case the reflector used is a perforated mirror attached to a spectacle frame.

Expertness in the use of the laryngoscope is chiefly the result of practice; nevertheless, the observance of certain precautions will materially assist the operator in the acquisition of dexterity.

Some patients require to be approached by degrees. A partial examination of the faucial region with a few repetitions soon gives to the parts increase of toleration that facilitates the process of a complete laryngoscopic observation.

In cases requiring *operative* procedure, it is well to learn the patient to use the depressor himself, or to acquire the art of holding the tongue in proper bounds with a napkin. In such cases the operator will find the advantage of ambidexterity. He should early learn to introduce the laryngeal mirror with either hand.

Before introducing the mirror, and after the patient is properly seated and the observer has taken his convenient position before his patient, the observer warms the reflecting surface of the mirror a few seconds over the chimney of the lamp, so that the moisture of the expired air will not be condensed upon it; and that he may not make it too hot, he should apply it to the back of his hand to test its temperature.

Considerable tact will be requisite in the introduction of the mirror, as a slight touching of the posterior surface of tongue will cause so much involuntary spasmodic action of the fauces as for the time to prevent any further manipulation. So too to avoid the uvula a little vocal trick is called into service—the vocalization of "ah," "oh," etc.—elevates momentarily the uvula when the operator slips the mirror in situ; carefully avoiding at the same time to tickle the pharynx.

The laryngeal mirror is not to be kept too long in the fauces. The observations are to be made promptly and briefly, and the patient permitted to rest, especially until he has become accustomed to the use of the instrument.

Of course, it will be readily understood that the laryngeal passages are inverted, and the observer must learn to accommodate himself to this reversed position of the structures in all operative processes. We close the present paper by giving views of the healthy larynx as shown by the laryngoscope. These illustrations are also copies from Bennett.

Fig. 2

Fig. 2 gives a view of the healthy larynx, when the vocal cords are closed as in sounding the high notes.

Fig. 3 is a view during ordinary breathing, and Fig. 4 gives a view during deep inspiration, the trachea straight, the glottis

Fig. 3.

Fig. 4.

widely dilated, and the rings of the trachea and bifurcation of the bronchi are seen through it.

My friend, Dr. Bruhl, has already contributed some interesting papers on these points in former numbers of this journal; nevertheless, in another paper I shall continue this subject and give some illustrations of those structures, as we see them in the more frequent forms of disease.—*Cincinnati Lancet.*

## Figures and Tables

**Figure f1:**
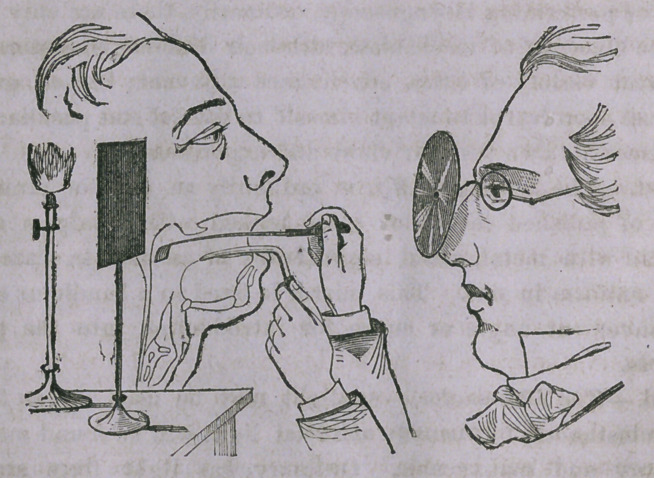


**Fig. 2 f2:**
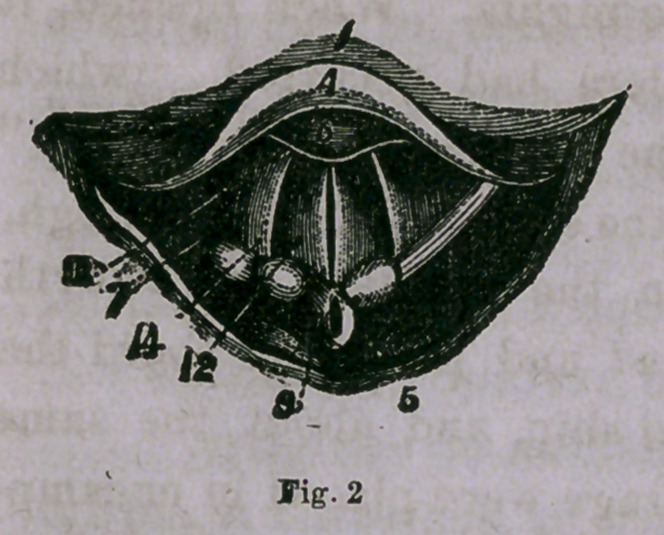


**Fig. 3. f3:**
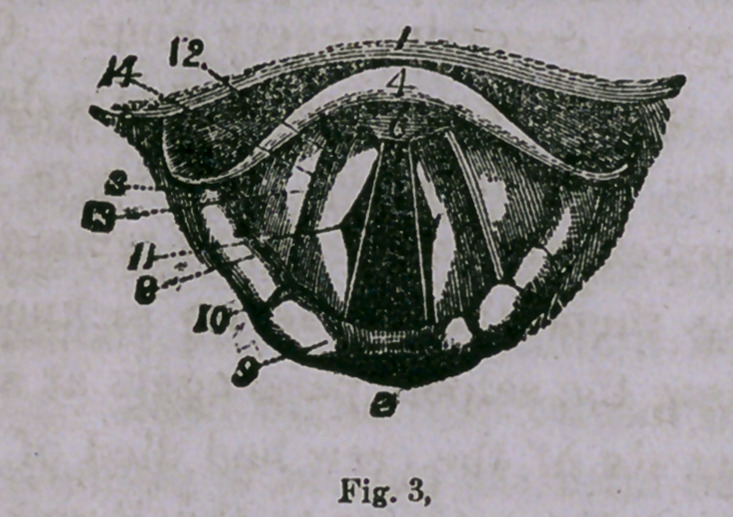


**Fig. 4. f4:**